# Supplementary material for: Bayesian hierarchical models for multivariate mixed responses with repeated measures: A case study in arterial occlusive disease
Source: PLoS One. 2026 Apr 15;21(4):e0346331. doi: 10.1371/journal.pone.0346331 (PMC13082732; doi:10.1371/journal.pone.0346331)
Supplement: S1 Table — (DOCX) [file pone.0346331.s001.docx]

Appendix Table 1: Posterior estimates with a variety of priors: Sensitivity analysis results

| Alternative priors | Parameter/  Covariates | Estimate (SD) | Median (50%) | 5%-95% HDP | Default prior Estimate (SD) | Percentage deviation |
| --- | --- | --- | --- | --- | --- | --- |
| Alternative prior I: Half-Cauchy (0,1) | *Intercept.*$Y_{1}$ | 0.1223 (0.083) | 0.1142 | -0.051, 0.2810 | 0.1324 (0.093) | -0.07628 |
|  | *Intercept.*$Y_{2}$ | -9.8925 (4.890) | -0.8741 | -23.731, -4.0254 | -9.8223 (4.883) | 0.007147 |
|  | Leg.$Y_{1}$ | 0.0213 (0.032) | 0.0165 | -0.0121, 0.0351 | 0.0234 (0.035) | -0.08974 |
|  | Leg.$Y_{2}$ | 1.1212 (0.970) | 1.0145 | -0.0162, 3.7654 | 1.1411 (0.965) | -0.01744 |
|  | *Ultrasound.*$Y_{1}$ | -0.0210 (0.121) | 0.0245 | -0.0112, 0.0345 | -0.0217 (0.122) | -0.03226 |
|  | *Ultrasound.*$Y_{2}$ | 12.5101 (5.720) | 11.2354 | 5.0102, 26.7801 | 12.5443 (5.711) | -0.00273 |
|  | *RCP.*$Y_{1}$ | 0.0210 (0.030) | 0.0235 | -0.0210, 0.0356 | 0.5801 (0.082) | **-0.9638** |
|  | *RCP.*$Y_{2}$ | 0.0211(0.020) | 0.1231 | -0.0112, 0.0871 | 0.0221(0.022) | -0.04525 |
|  | *Level 2*: $Y_{1}$ | 0.1211 | 0.1210 | 0.0571, 0.1762 | 1.9212 | **-0.93697** |
|  | *Level 1:* $Y_{2}$ | 0.3810 | 0.3101 | 0.0153, 1.0854 | 1.3032 | -0.70764 |
|  | *Level 1:* Leg | 0.9210 | 0.8452 | 0.0765, 2.4761 | 0.9425 | -0.02281 |
|  | *Cor(*$Y_{1},Y_{2}$*):*$\rho_{y_{1},y_{2}}$ | 0.0801 | 0.1436 | -0.4201, 0.5632 | 0.0401 | **0.997506** |
|  | Parameter | Estimate (SD) | Median (50%) | 5%-95% HDP | Default prior Estimate(SD) | Percentage deviation |
| Alternative prior II: Normal (0, 5) | *Intercept.*$Y_{1}$ | 0.1301 (0.067) | 0.1243 | -0.1415, 0.3221 | 0.1423 (0.077) | -0.08573 |
|  | *Intercept.*$Y_{2}$ | -0.2010 (0.111) | -0.1143 | -0.1514, -0.0855 | -0.2133 (0.211) | -0.05767 |
|  | Leg.$Y_{1}$ | -0.0011 (0.001) | -0.0034 | -0.0124, 0.0015 | -0.0014 (0.003) | -0.21429 |
|  | Leg.$Y_{2}$ | 0.0551 (0.032) | 0.1241 | -0.2260, 0.4841 | 0.0651 (0.042) | -0.15361 |
|  | *Ultrasound.*$Y_{1}$ | -0.0120 (0.012) | -0.0213 | -0.0113, 0.0423 | -0.0211 (0.021) | -0.43128 |
|  | *Ultrasound.*$Y_{2}$ | 0.9461(0.213) | 0.5461 | 0.1125, 1.2301 | 0.8742 (0.203) | 0.082247 |
|  | *RCP.*$Y_{1}$ | 0.0012(0.002) | 0.0011 | 0.0011, 0.6401 | 0.0131 (0.012) | **-0.9084** |
|  | *RCP.*$Y_{2}$ | 0.0011(0.003) | 0.0212 | 0.0002, 1.2013 | 0.0012(0.014) | -0.08333 |
|  | *Level 2*: $Y_{1}$ | 0.0142 | 1.2451 | 0.0122, 1.1012 | 1.3032 | -0.9891 |
|  | *Level 1:* $Y_{2}$ | 0.3871 | 0.0201 | 0.0123, 0.6624 | 0.0425 | 0.10823 |
|  | *Level 1:* Leg | 0.0316 | 0.0311 | 0.0212, 0.1743 | 0.0401 | -0.21197 |
|  | *Cor(*$Y_{1},Y_{2}$*):*$\rho_{y_{1},y_{2}}$ | 0.1024 | 0.1012 | -0.2044, 0.9201 | 0.1031 | -0.00679 |

***Note:*** The relative percentage deviation can be computed as: [((estimate using new alternative prior)-(estimate using default/reference prior))/ (estimate using default/reference prior)]*100. Interpreting percentage deviation results is largely subjective and depends on the metric used for comparing the parameters. However, a percentage deviation of less than 10% would likely be considered negligible [81]. Default estimate = posterior estimate (mean) of analysis with the BRMS default/ reference prior (*Student's t (3*); 5%-95% is the highest posterior density (HPD) interval.
