# Supplementary material for: Bayesian hierarchical models for multivariate mixed responses with repeated measures: A case study in arterial occlusive disease
Source: PLoS One. 2026 Apr 15;21(4):e0346331. doi: 10.1371/journal.pone.0346331 (PMC13082732; doi:10.1371/journal.pone.0346331)
Supplement: Attachment — Submitted filename: Reviewer commentsPONE-D-25-42936.docx [file pone.0346331.s002.docx]

The paper by Endris and Ali entitled “Bayesian Hierarchical Models for Multivariate Mixed Responses with Repeated Measures: A Case Study in Arterial Occlusive Disease” develops and applies Bayesian hierarchical models that jointly analyze repeated binary (health status) and continuous (disease severity score) leg-level outcomes for arterial occlusive disease, comparing Metropolis–Hastings, Gibbs and Hamiltonian Monte Carlo (HMC) fitting strategies and a set of model-selection criteria (DIC, ICOMP, WAIC, LOO, K-fold). The study's main contribution is applied: it argues that a full random-intercepts-and-slopes specification estimated via HMC gives the best fit for this small clinical dataset, but important methodological, reporting, and inference problems limit the paper’s current suitability for publication.

In general, the dataset is very small and poorly described; the manuscript lacks full model specification (especially priors and likelihood details), has internal inconsistencies across tables/figures and methods, and uses Bayesian diagnostics and frequentist language interchangeably (p-values), which together prevent reliable assessment of the claims. The article at this stage suffers from many issues that need to be addressed before a decision can be made. The linearity and clarity of the exposition should be reviewed. Below are some comments for the authors to address:

**Major Comments**

1. **Data description & sample size (fatal for claims of generalizability):**

The manuscript states “*16 patients ... assessed four times on the upper and lower sides of their left and right legs*.” This wording is ambiguous: is the total number of observations 16×4 (64) measurements, or 16 patients × 4 sides × 4 occasions = 256? The numbers in the tables (ESS, levels) suggest small effective sample sizes. Small-sample behavior of WAIC/LOO/DIC and HMC can be problematic; author claims that HMC “*consistently outperformed*” others require simulation or at least sensitivity analysis to sample-size. Please state clear counts: N (patients), number of legs per patient, number of timepoints/occasions, and total observations for each response. Report any exclusions. Also report ethical approvals and date ranges explicitly (data are 1988/89 - that should be explicit in Methods and discussed as old data).

In addition, add a clear table “Data summary” (N patients, legs, repeats per leg, missingness per variable). Re-run key model checks stratified by patients and by leg if possible. If sample size is too small to reliably estimate full random slopes, tone down claims or add simulations (see point 4).

2. **Model specification incomplete: priors, link/latent model for binary, and joint likelihood missing.**

The manuscript frequently describes the idea in words but never gives full probabilistic model notation: what is the joint model for (Y1, Y2)? Is the binary modeled via logistic (logit) or probit? Do the authors model the binary with a latent continuous variable to allow joint normal random effects? If so, that must be written explicitly, with equations for likelihood and all priors. Which parameters have random slopes and at which levels? How are cross-outcome covariances of random effects parameterized?

Consider adding a compact mathematical model block (likelihoods for each response, linear predictors, random-effect structure, prior distributions). Report all priors (including hyperpriors for variance components and correlation priors). For HMC/brms runs include the brms formula code (or Stan code) in Supplementary material.

3. **Priors and sensitivity:** No priors are specified; yet Bayesian inference depends on priors, especially with small N. The authors must report priors for fixed effects, variance components, and correlation/covariance matrices. Provide prior sensitivity checks (at least one alternative weakly informative and one regularizing prior) and show how posterior inferences (especially random effects variances and fixed effects of interest like ultrasound) change.
4. **Claims about HMC superiority are over-stated without simulation evidence.**

HMC often mixes better for hierarchical models (<https://doi.org/10.48550/arXiv.1312.0906> ; <https://doi.org/10.48550/arXiv.1507.04544>), but performance depends on parameterization, marginalization, and data size. With such a small dataset, differences in information criteria may be due to implementation differences (how WAIC/LOO are approximated, number of iterations, warmup, adaptation settings) rather than intrinsic algorithm superiority. The authors should either (a) add a small simulation study that mimics the observed data (N, clustering, effect sizes) showing HMC recovers parameters more reliably than MH/Gibbs under realistic priors and model misspecification, **or** (b) substantially tone down the claim and present HMC as *one* effective option, with caveats. See the references mentioned above.

5. **Inconsistent/incorrect reporting of Bayesian inference and use of p-values.**

The manuscript reports Z, Pr(>|z|), pMCMC, and “** significant at 5%”* across the tables. These are frequentist summaries or hybrid outputs and are not standard Bayesian summaries. For Bayesian output you should report posterior mean/median, 95% credible intervals, and posterior probabilities (P(β>0 | data)). If the software produces pMCMC (proportion of posterior beyond zero), call it “posterior probability” not p-value. Remove or clarify the use of z-tests unless justified (large-sample normal approximation of posterior?).

6. **Model comparison details and reproducibility.**

It is unclear how WAIC/LOO/K-fold were computed for each package (R2MLwiN, MCMCglmm, brms). Non-factorized models and models with random effects require special handling for LOO/WAIC (see <https://doi.org/10.1007/s11222-016-9696-4>). The manuscript must say exactly which implementation (package, version) and the exact commands or code used for criteria. Were PSIS diagnostics checked (k-hat values)? K-fold CV should include splits and seed. DIC is known to be unreliable for hierarchical models. So, justify including it.

Consider adding code snippets or appendix with exact commands and the versions. Report PSIS k-diagnostics for LOO.

7. **Numerical issues/table inconsistencies and impossible values.**

Several reported numbers and inconsistencies are concerning: Table 3.1 shows large positive Ultrasound coefficients (4.89) with CI 4.51–9.254 — but Table 3.3 HMC reports Ultrasound Y1 ≈ 0.28 (CI 0.08–0.48). The signs and magnitudes vary across methods far beyond credible intervals. That suggests differences in parameterization, scaling of covariates, or errors in table labeling. Also negative σ_ultrasound of −5.425 (Table 3.1) is impossible for a standard deviation, likely a log-scale parameter or formatting error. Check and confirm.

Number tables according to the PLOS ONE guideline, not as a book chapter or report.

Re-check all tables, standardize variable scaling, explicitly report whether predictors were standardized. Remove or explain impossible negative SDs. Provide posterior density plots for key parameters across methods to show consistency/inconsistency.

8. **Diagnostics & posterior predictive checks (PPC) are superficial.**

The paper refers to Figures 3.3–3.7 but there is no informative description of posterior predictive checks, calibration, or residual patterns. Bayesian model assessment must include PPCs tailored to each outcome type (binary outcome calibration plots, ROC/AUC where relevant, continuous residuals). Provide prior/posterior checks, PPC plots and discussion of misfit if any.

Consider including PPC plots (observed vs replicated summary statistics), calibration curves for binary outcome, and a short paragraph interpreting them.

9. **Use of outdated dataset & clinical context.**

Data were collected in 1988/89: clinical practice and measurement protocols have changed. Discuss this limitation explicitly and do not overclaim clinical implications. Add modern references about ultrasound and cuff pressure diagnostic value for PAD. See <https://doi.org/10.7863/jum.1992.11.3.95> ; and <https://www.ncbi.nlm.nih.gov/books/NBK576430/>

10. **Reproducibility: code and data availability.**

If possible, the manuscript must provide code (Stan/brms/MCMCglmm scripts) and, if patient privacy allows, the data or a simulated dataset reproducing modelling steps. Without code, reviewers cannot verify HW results. Consider adding a GitHub/Zenodo link to code and (if possible) data; if data cannot be shared, provide a simulated example with identical dimensions and signal/noise to reproduce main findings.

11. Ethical Statements: Please, reconsider this statement. Even if your study uses secondary, anonymized, or historical data, most journals require an explicit ethics statement clarifying the ethical status of the data. This ensures transparency and compliance with institutional and journal policies.

**Additional comments**

(i) Terminology & typos: “Metropolis-Hastening” (multiple occurrences) or “Metropolis–Hastings” ? “random gradient” vs “random slope”, be consistent. “null (empty) model” is okay but use consistent term (intercept-only). Many grammatical errors and awkward sentences throughout: a thorough language edit is needed.
(ii) Figures & Table formatting: Figures and Tables should be referenced following the journal guidelines. Ensure high-quality figures with captions that describe data plotted and the sample size; tables should include exact definitions of columns (what is ESS, Bulk/Tail ESS, R-hat). Use consistent number of digits.
(iii) If possible, use posterior probabilities or Bayes factors if you want to indicate strength of evidence instead of asterisks for Bayesian credible intervals.
(iv) Matrix notation in Methods mixes many symbols and is hard to read. Provide a short notation table (variables, levels).
(v) Abstract: mention the software used explicitly.

What do you mean by null random intercepts? “models without random effects” or “intercept-only random effects”?

Avoid absolute claims like “yields the most reliable inference” — instead say “provided better performance on the considered information criteria for this dataset” and mention the dataset is small and historical.

Add brief numbers: sample size (N patients, observations).

Remove p-value-style claims in abstract.

(vi) Introduction is well written. But consider structuring it following: (a) joint modeling literature and why joint models help, (b) computational challenges and why algorithm comparison matters, (c) data context and gap the paper fills.

Remove repeated or redundant paragraphs (many paragraphs restate same idea).

Cite and discuss more recent multivariate Bayesian joint-model literature to show novelty <https://doi.org/10.1146/annurev-statistics-112723-034334> <https://doi.org/10.1186/s12874-024-02333-z>

(vii) Methods could be restructured considering (1) the Model specification (explicit mathematical formulation), (2) Priors (Add priors for β (Normal(0, 5)?), for σ (half-Student-t or half-Cauchy?), for correlation matrices (LKJ prior with parameter), and justify choices.)), (3) MCMC & software (software, package versions, and exact settings. For HMC, specify whether models were parameterized centered or non-centered), (4) Model comparison (Explain how WAIC, LOO, K-fold CV, DIC, and ICOMP were computed and which packages computed each. For LOO, report PSIS k estimates and how problematic observations were handled), and (5) Data.
(viii) Results could be organized around Descriptives, Fitted models, Model comparison, and Diagnostic and PPC.
(ix) Discussion and conclusions can be reworked to avoid overstatement. The dataset is small/old, conclusions about clinical practice should be tentative. Emphasize methodological lessons (how HMC handled hierarchical correlation structure when properly parameterized) but include caveats about priors and sample size. Suggest future directions.
(x) For references, add canonical methodological references:

<https://doi.org/10.1186/s12874-024-02333-z> <https://doi.org/10.1146/annurev-statistics-112723-034334> <https://doi.org/10.48550/arXiv.1206.1901> <https://doi.org/10.48550/arXiv.1312.0906> <https://doi.org/10.1007/s11222-016-9696-4>

At present the manuscript cannot be recommended for publication due to the reasons mentioned above. If the authors address the major issues above (particularly full model specification, priors, reproducible code, and consistent results or simulations that explain method differences) and tone down any overstatements about clinical implications, the manuscript could make a useful applied-methods contribution illustrating algorithmic comparisons for joint mixed outcomes. I look forward to see the revised version of this paper.
