# Supplementary material for: Bayesian hierarchical models for multivariate mixed responses with repeated measures: A case study in arterial occlusive disease
Source: PLoS One. 2026 Apr 15;21(4):e0346331. doi: 10.1371/journal.pone.0346331 (PMC13082732; doi:10.1371/journal.pone.0346331)
Supplement: Attachment — Submitted filename: Response to Reviewers.docx [file pone.0346331.s004.docx]

**Journal Requirements: Editorial Comment – Formatting and File Naming**

1. Please ensure that your manuscript meets PLOS ONE's style requirements, including those for file naming. The PLOS ONE style templates can be found at

<https://journals.plos.org/plosone/s/file?id=wjVg/PLOSOne_formatting_sample_main_body.pdf> and

[https://journals.plos.org/plosone/s/file?id=ba62/PLOSOne_formatting_sample_title_authors_affiliations.pdf](https://track.editorialmanager.com/CL0/https:%2F%2Fjournals.plos.org%2Fplosone%2Fs%2Ffile%3Fid=ba62%2FPLOSOne_formatting_sample_title_authors_affiliations.pdf/1/010f019a2ea3ebdd-fb927e6c-b549-4895-8c73-49da977d4f8d-000000/HnzqrA19ex_xa5JC7LqG2bf7e5o2ehPSqnqNtJtEzuI=235)

***Response:***  *Thank you for the reminder. We have carefully reviewed and revised our manuscript to ensure full compliance with the PLOS ONE formatting and file naming guidelines. The revised submission adheres to the structure outlined in the official style templates, and all files have been renamed in accordance with the journal's specifications.*

2. Please note that PLOS One has specific guidelines on code sharing for submissions in which author-generated code underpins the findings in the manuscript. In these cases, all author-generated code must be made available without restrictions upon publication of the work. Please review our guidelines at <https://journals.plos.org/plosone/s/materials-and-software-sharing#loc-sharing-code> and ensure that your code is shared in a way that follows best practice and facilitates reproducibility and reuse.

***Response:***

*We acknowledge and fully support PLOS ONE's policy on code sharing. To facilitate reproducibility and reuse, all author-generated code used in the manuscript will be made available* ***without restriction*** ***upon acceptance/ publication****. The code will be sent as supplementary material in the final version of the manuscript and will be linked (published) at* [**https://doi.org/10.5281/zenodo.17621419**](https://doi.org/10.5281/zenodo.17621419)**.**

*In the meantime, the code is available upon request from the corresponding author via email at* [*end384@gmail.com*](mailto:end384@gmail.com)*. This is due to the project being continuous with the subsequent two phases, which involve related code or data usage.*

3. We note that the grant information you provided in the 'Funding Information' and 'Financial Disclosure' sections do not match.

When you resubmit, please ensure that you provide the correct grant numbers for the awards you received for your study in the 'Funding Information' section.

**Response:**

*The correct funding information should be stated as: "This work was supported and funded by the Deanship of Scientific Research at Imam Mohammad Ibn Saud Islamic University (IMSIU) (****grant number IMSIU-DDRSP2501****)."*

4. Thank you for stating the following financial disclosure:

 "This study was supported by Department of Mathematics and Statistics, College of Science, Imam Mohammad Ibn Saud Islamic University (IMSIU), Riyadh, Saudi Arabia"

Please state what role the funders took in the study.  If the funders had no role, please state: "The funders had no role in study design, data collection and analysis, decision to publish, or preparation of the manuscript."

If this statement is not correct you must amend it as needed.

Please include this amended Role of Funder statement in your cover letter; we will change the online submission form on your behalf.

**Response:**

***Response (to be included in the cover letter):***

*This work was supported and funded by the Deanship of Scientific Research at Imam Mohammad Ibn Saud Islamic University (IMSIU) (grant number IMSIU-DDRSP2501). We (authors) declare that t****he funders had no role in the study design, data collection and analysis, the decision to publish, or the preparation of the manuscript.***

5. We noted in your submission details that a portion of your manuscript may have been presented or published elsewhere. "This study used open-access data. Data were collected at Broadgreen Hospital in Liverpool, England, in 1988/89 (Percy, 1992). <https://doi.org/10.2307/2532336>" Please clarify whether this [conference proceeding or publication] was peer-reviewed and formally published. If this work was previously peer-reviewed and published, in the cover letter please provide the reason that this work does not constitute dual publication and should be included in the current manuscript.

**Response:**

We thank the editor for the clarification.

***Response (to be included in the cover letter):***

This study used open-access data. Data were collected at Broadgreen Hospital in Liverpool, England, in 1988/89 (Percy, 1992). <https://doi.org/10.2307/2532336>". *This data source work was previously peer-reviewed and published. Using open-access data from a previously published paper does not constitute dual publication* ***because our new manuscript presents a secondary analysis with a different research question, presents new analyses, focuses on a different aspect, or has a new purpose, and the previous publication is cited correctly, rather than republishing the same findings and conclusions.***

6. Please note that funding information should not appear in any section or other areas of your manuscript. We will only publish funding information present in the Funding Statement section of the online submission form. Please remove any funding-related text from the manuscript.

**Response:**

*We thank the editor for the clarification. We will remove any funding-related text from the manuscript.*

7. Thank you for uploading your study's underlying data set. Unfortunately, the repository you have noted in your Data Availability statement does not qualify as an acceptable data repository according to PLOS's standards.

At this time, please upload the minimal data set necessary to replicate your study's findings to a stable, public repository (such as figshare or Dryad) and provide us with the relevant URLs, DOIs, or accession numbers that may be used to access these data. For a list of recommended repositories and additional information on PLOS standards for data deposition, please see [https://journals.plos.org/plosone/s/recommended-repositories](https://track.editorialmanager.com/CL0/https:%2F%2Fjournals.plos.org%2Fplosone%2Fs%2Frecommended-repositories/1/010f019a2ea3ebdd-fb927e6c-b549-4895-8c73-49da977d4f8d-000000/szD9IMCXLSr8_s13rtCAQ73_8XWqb_2fGQ9Bcj8yu5Q=235).

**Response:**

We thank the editor for the clarification. In compliance with PLOS ONE's data availability policy, we have uploaded the minimal dataset necessary to replicate our findings to a stable public repository. The data used and analyzed during the current study are publicly available on Zenodo at the following DOI: <https://doi.org/10.5281/zenodo.17621089>

The Data Availability Statement in the manuscript has also been updated to reflect this change.

8. If the reviewer comments include a recommendation to cite specific previously published works, please review and evaluate these publications to determine whether they are relevant and should be cited. There is no requirement to cite these works unless the editor has indicated otherwise.

**Response:**

It is okay; reviewer #2 is suggested to include or cite some appropriate published works, and we have included a few of them as indicated in the updated version.

***Response to Reviewer #1***

**Reviewer #1: Major Points for Revision:**

1. Clarity of Model Specification: The mathematical description of the Bayesian hierarchical model for mixed (binary and continuous) responses needs significant improvement. While the general form is presented, the specific likelihoods, the way the two sub-models (for binary and continuous outcomes) are linked, and the full specification of the prior distributions are not sufficiently clear. Please provide a more detailed and unambiguous mathematical formulation of the final model used.

This should include: The precise likelihood functions for both the continuous and binary responses.
A clear definition of all parameters, including fixed effects, random effects (intercepts and slopes), and variance components.

The specific prior distributions used for all parameters (e.g., regression coefficients, variance-covariance matrices for random effects). Justifying the choice of these priors (e.g., non-informative, weakly informative) is crucial for the reproducibility and interpretation of a Bayesian analysis.

**Response:**

We sincerely thank the reviewer(s) for raising these important points regarding the model specification and related issues. We agree. In the revised manuscript, we include a full probabilistic model specification, showing the likelihood for each outcome, the joint model structure, and the priors for each MCMC approach in R packages. The mathematical description of the Bayesian hierarchical model for mixed (binary and continuous) responses is updated and improved in the revised manuscript, with likelihood, prior, and posterior definitions incorporating parameters as fixed effects, random effects, and variance components (Section 2.3 (Methods).. The models were fitted with default priors, which serve as a standard weakly informative prior in each MCMC approach. In addition, due to HMC in R-BRMS's flexibility in prior specification and choice using alternative weakly informative or regularized priors, changes with Half-Cauchy (0,1) for scale parameters and Normal (0, 5) for fixed effects were compared by using prior predictive checks (Fig setting sample_prior = "only"), and sensitivity analysis (revised manuscript Appendix table )

**Reviewer #1: Major Points for Revision:**

2. Reporting of MCMC Diagnostics: A critical component of any Bayesian analysis is demonstrating the convergence of the MCMC chains. The manuscript currently lacks a detailed report of convergence diagnostics. Please add a section or supplement that includes:
   Trace plots for key parameters to visually inspect mixing and stationarity.
   Gelman-Rubin diagnostic statistics (R) for all major parameters to quantitatively assess convergence. Values should be close to 1.0.

A report on the effective sample sizes (ESS) to ensure that the posterior distributions are adequately characterized.

**Response:**

Evaluation of MCMC convergence Diagnostics and conditional/marginal effects for the best-fitted model is included and updated in section 3.4, with a detailed explanation of ESS, R hat, and other convergence diagnostics. Effective sample size (ESS) measures the number of independent samples a Markov chain Monte Carlo (MCMC) sampler would need to produce the same precision as the current autocorrelated sample. The Metropolis-Hastings ESS is sensitive to the variance of the proposal distribution; high autocorrelation leads to a low ESS. Hamiltonian Monte Carlo (HMC) and its variants generally have higher ESS than basic Metropolis-Hastings because their gradient-based updates allow for more efficient exploration of parameter space. However, it is still affected by adaptation, tuning, and the model's specific formulation. Gibbs samplers can achieve high ESS if the conditional distributions are easy to sample from and the variables are not highly correlated.  Different MCMC samplers yield different ESS values because of their distinct mechanisms for exploring the posterior distribution. Hamiltonian Monte Carlo (HMC) generally yields higher ESS per iteration in many complex problems, whereas Metropolis-Hastings (MH) and Gibbs samplers can be less efficient, especially in high-dimensional or highly correlated posteriors(Gamerman & Lopes, 2006).

Although R-hat = 1.0 is the ideal value and indicates perfect convergence across all MCMC chains, R-hat statistic, or potential scale reduction factor, values < 1.1 indicate chain convergence, while values > 1.1 are indicative of less mixing and stationarity. For Hamiltonian Monte Carlo (HMC) and other MCMC methods, an R-hat value of 1.01 indicates excellent convergence. However, a value slightly higher than 1.01 might still be acceptable, with 1.1 being a standard threshold for early stages. R-hat values significantly greater than 1.00 suggest that the Markov chains have not mixed well or converged to the target distribution, with the highest acceptable value often considered to be around 1.01 for robust convergence.  The recommended threshold for an R-hat value of 1.01 or less is a modern standard used by probabilistic programming software such as Stan (Gelman & Rubin, 1992; Gelman & Shirley, 2011). Others propose improvements to the traditional R-hat and recommend the ESS > 400 threshold for reliable diagnostics, which is the standard used in STAN (Vehtarh et al., 2021). However, our estimate of R hat (1.01) is approximated as 1.00 in the revised manuscript.

**Reviewer #1: Major Points for Revision:**

3. Interpretation of the Case Study Results: The results section primarily focuses on model comparison metrics (DIC, WAIC, LOO-IC, etc.), concluding that the random intercepts and slopes model estimated via HMC is superior. While this is an important methodological finding, the paper falls short in interpreting the clinical or practical meaning of the parameter estimates from this best-fitting model. The manuscript would be substantially strengthened by a discussion of:

Which predictors were significantly associated with the binary and continuous outcomes?
What is the magnitude and direction of these effects?
What do the random effects variances tell us about the heterogeneity between subjects in their disease progression trajectories?

A table presenting the posterior means and 95% credible intervals for the fixed and random effect parameters from the selected model is essential. Without this, the case study serves only as a vehicle for the methodological comparison, missing an opportunity to provide substantive insights into Arterial Occlusive Disease.

**Response:**

We sincerely thank the reviewer for raising these important points. The marginal effects of each fixed and random term in the best-fit model were presented and reported in Figures 6 and 7. Moreover, Table 8 presents the posterior means and 95% credible intervals for the fixed and random effect parameters. However, in the revised manuscript, a detailed report and interpretations on fixed and random effects were included next to Table 8. Posterior mean estimates with median 95% credible interval posterior density (HPD) using the default prior and two alternative priors were reported and discussed in the Appendix.

**Reviewer #1: Major Points for Revision:**

4. Justification for Ethics Statement: The authors state "No need of Ethical Statements". However, the study uses patient data. Although the data is from a publicly available source, it is standard practice to state that the original study from which the data were obtained received ethical approval and that the current analysis uses anonymized, de-identified data. Please revise the ethics statement accordingly.

**Response:**

We sincerely thank the reviewer for raising this important point of the Ethical statement. However, we thought this study is a modeling application using secondary data from a peer-reviewed, open-access paper. We understand the reviewer's suggestion and have updated it in the revised manuscript as part 2.4.

**Reviewer #1: Minor Points:**

Please provide more details about the Arterial Occlusive Disease (AOD) dataset in the Methods section. Include the number of patients, the number of repeated measurements per patient, and a clear description of the specific binary and continuous outcome variables and the predictor variables used in the model.
The introduction contains some repetitive phrasing and could be made more concise while still motivating the study effectively.
Ensure all abbreviations are defined at their first use, in addition to the list provided at the end of the manuscript.

**Response:**

Thank you, dear reviewer 1. A detailed description and summary structure of the Arterial Occlusive Disease (AOD) dataset are provided. All abbreviations are defined at their first use.

**Response to Reviewer #2**

**Reviewer #2:**

The paper by Endris and Ali entitled "Bayesian Hierarchical Models for Multivariate Mixed Responses with Repeated Measures: A Case Study in Arterial Occlusive Disease" develops and applies Bayesian hierarchical models that jointly analyze repeated binary (health status) and continuous (disease severity score) leg-level outcomes for arterial occlusive disease, comparing Metropolis–Hastings, Gibbs and Hamiltonian Monte Carlo (HMC) fitting strategies and a set of model-selection criteria (DIC, ICOMP, WAIC, LOO, K-fold). The study's main contribution is applied: it argues that a full random-intercepts-and-slopes specification estimated via HMC gives the best fit for this small clinical dataset, but important methodological, reporting, and inference problems limit the paper's current suitability for publication.

In general, the dataset is very small and poorly described; the manuscript lacks full model specification (especially priors and likelihood details), has internal inconsistencies across tables/figures and methods, and uses Bayesian diagnostics and frequentist language interchangeably (p-values), which together prevent reliable assessment of the claims. The article at this stage suffers from many issues that need to be addressed before a decision can be made. The linearity and clarity of the exposition should be reviewed. Below are some comments for the authors to address:

**Reviewer #2: Major Comments**

1. **Data description & sample size (fatal for claims of generalizability):**

The manuscript states "*16 patients ... assessed four times on the upper and lower sides of their left and right legs*." This wording is ambiguous: is the total number of observations 16×4 (64) measurements, or 16 patients × 4 sides × 4 occasions = 256? The numbers in the tables (ESS, levels) suggest small effective sample sizes. Small-sample behavior of WAIC/LOO/DIC and HMC can be problematic; author claims that HMC "*consistently outperformed*" others require simulation or at least sensitivity analysis to sample-size. Please state clear counts: N (patients), number of legs per patient, number of timepoints/occasions, and total observations for each response. Report any exclusions. Also report ethical approvals and date ranges explicitly (data are 1988/89 - that should be explicit in Methods and discussed as old data).

In addition, add a clear table "Data summary" (N patients, legs, repeats per leg, missingness per variable). Re-run key model checks stratified by patients and by leg if possible. If sample size is too small to reliably estimate full random slopes, tone down claims or add simulations (see point 4).

**Response:**

A detailed description and summary structure of the Arterial Occlusive Disease (AOD) dataset are provided in the revised manuscript. ESS estimates are based on MCMC draws generated by the algorithms. Bulk Effective Samples Size (ESS) that indicate posterior means and medians, and Tail Effective Samples Size (ESS) indicating posterior variances and tail quantiles obtained in HMC.The threshold value of ESS among different MCMC approaches was explained and described in the revised manuscript.

2. **Model specification incomplete: priors, link/latent model for binary, and joint likelihood missing.**

The manuscript frequently describes the idea in words but never gives full probabilistic model notation: what is the joint model for (Y1, Y2)? Is the binary modeled via logistic (logit) or probit? Do the authors model the binary with a latent continuous variable to allow joint normal random effects? If so, that must be written explicitly, with equations for likelihood and all priors. Which parameters have random slopes and at which levels? How are cross-outcome covariances of random effects parameterized?

Consider adding a compact mathematical model block (likelihoods for each response, linear predictors, random-effect structure, prior distributions). Report all priors (including hyperpriors for variance components and correlation priors). For HMC/brms runs include the brms formula code (or Stan code) in Supplementary material.

**Response:**

We sincerely thank the reviewer(s) for raising these important points regarding the model specification and related issues. We agree. In the revised manuscript, we include a full probabilistic model specification, showing the likelihood for each outcome, the joint model structure, and the priors for each MCMC approach in R packages. The mathematical description of the Bayesian hierarchical model for mixed (binary and continuous) responses is updated and improved in the revised manuscript, with likelihood, prior, and posterior definitions incorporating parameters as fixed effects, random effects, and variance components (Section 2.3 (Methods). The models were fitted with default priors, which serve as a standard weakly informative prior in each MCMC approach. In addition, due to HMC in R-BRMS's flexibility in prior specification and choice using alternative weakly informative or regularized priors, changes with Half-Cauchy (0,1) for scale parameters and Normal (0, 5) for fixed effects were compared by using prior predictive checks (Fig. setting sample_prior = "only"), and sensitivity analysis (revised manuscript Appendix table )

3. **Priors and sensitivity:** No priors are specified; yet Bayesian inference depends on priors, especially with small N. The authors must report priors for fixed effects, variance components, and correlation/covariance matrices. Provide prior sensitivity checks (at least one alternative weakly informative and one regularizing prior) and show how posterior inferences (especially random effects variances and fixed effects of interest like ultrasound) change.

**Response:**

For each model specification, priors for each parameter in the corresponding R package were specified. A prior sensitivity analysis is performed; we re-estimate the best-fitting model with different priors and compare the posterior inferences. Comparing these results shows how sensitive posterior estimates of random-effect variances are (Appendix added).

4. **Claims about HMC superiority are over-stated without simulation evidence.**

HMC often mixes better for hierarchical models (<https://doi.org/10.48550/arXiv.1312.0906> ; <https://doi.org/10.48550/arXiv.1507.04544>), but performance depends on parameterization, marginalization, and data size. With such a small dataset, differences in information criteria may be due to implementation differences (how WAIC/LOO are approximated, number of iterations, warmup, adaptation settings) rather than intrinsic algorithm superiority. The authors should either (a) add a small simulation study that mimics the observed data (N, clustering, effect sizes) showing HMC recovers parameters more reliably than MH/Gibbs under realistic priors and model misspecification, **or** (b) substantially tone down the claim and present HMC as *one* effective option, with caveats. See the references mentioned above.

**Response:**

Thank you, dear reviewer #2. Besides the small size of the dataset, different MCMC approaches use different parameterizations. That might result in different outputs. We considerably tone down the inferences made and present HMC as optional, effective only with model parametrization, MCMC draws, parameter scaling, and other limited structures.

5. **Inconsistent/incorrect reporting of Bayesian inference and use of p-values.**

The manuscript reports Z, Pr(>|z|), pMCMC, and "** significant at 5%"* across the tables. These are frequentist summaries or hybrid outputs and are not standard Bayesian summaries. For Bayesian output you should report posterior mean/median, 95% credible intervals, and posterior probabilities (P(β>0 | data)). If the software produces pMCMC (proportion of posterior beyond zero), call it "posterior probability" not p-value. Remove or clarify the use of z-tests unless justified (large-sample normal approximation of posterior?).

**Response:**

The R2MLwiN package employs a hybrid approach that can generate both frequentist and Bayesian-style output, serving as an interface to the standalone software MLwiN, which provides both estimation methods: Iterative Generalized Least Squares (IGLS) for maximum likelihood and Markov Chain Monte Carlo (MCMC) for Bayesian inference. As explained b R2MLwiN offers the option to display the Z-score and a corresponding two-tailed P-value, but this relies on the assumption that the posterior distribution is approximately normal.

MCMCglmm is a fully Bayesian package designed to fit Generalized Linear Mixed Models using MCMC methods. In a strict Bayesian framework, researchers typically use credible intervals (which represent the probability that the true parameter value lies within a given range) or posterior probabilities (e.g., the proportion of posterior samples that are above or below zero) to make inferences, rather than P-values or Z-scores. The Z and P values provided in the MCMCglmm output are convenient frequentist approximations for the fixed effects. The Z-statistic is typically calculated as the posterior mean divided by the posterior standard error (SD). The P-value is then derived from this Z-statistic using the standard normal distribution.

Hadfield (2010) clarifies in a forum post that the default P-value (pMCMC) in the output is a one-sided Bayesian P-value (the proportion of the MCMC chain with the opposite sign to the point estimate), not a traditional frequentist P-value. Z is a test statistic analogous to a Wald Z-statistic in frequentist models, calculated as the posterior mean divided by the posterior standard deviation. This is considered a large-sample normal approximation of the posterior mean. Pr(>|z|) is the p-value associated with the Z-statistic, used for hypothesis testing in MCMCglmm and HM in a frequentist manner. The *pMCMC* is a Bayesian measure, defined as twice the proportion of the posterior distribution that is of the same sign as the mean (or equivalently, the smallest posterior tail probability). It is a Bayesian analogue of a "p-value". The pMCMC value is an attempt to create a Bayesian analogue to the frequentist two-tailed p-value. While it uses the posterior distribution (a Bayesian concept), interpreting it as a standard p-value can be problematic and lead to misinterpretations. The package author has noted it's "not a p-value as such". The "pMCMC" is expressed as "Posterior Probability". The significance of the posterior mean estimate ("* significant at 5%") was assessed using the 95% credible interval.

6. **Model comparison details and reproducibility.**

It is unclear how WAIC/LOO/K-fold were computed for each package (R2MLwiN, MCMCglmm, brms). Non-factorized models and models with random effects require special handling for LOO/WAIC (see <https://doi.org/10.1007/s11222-016-9696-4>). The manuscript must say exactly which implementation (package, version) and the exact commands or code used for criteria. Were PSIS diagnostics checked (k-hat values)? K-fold CV should include splits and seed. DIC is known to be unreliable for hierarchical models. So, justify including it.

Consider adding code snippets or an appendix with exact commands and the versions. Report PSIS k-diagnostics for LOO.

**Response:**

The computation of WAIC, LOO, and K-fold differs significantly across the R2MLwiN, MCMCglmm, and brms packages, largely due to their underlying estimation methods and their integration with external packages (such as the loo package).

**brms**

The brms package is built to work seamlessly with the loo package, which provides robust and dedicated functions for approximate Bayesian cross-validation.  WAIC and LOO: These are typically computed using the waic() and loo() functions, respectively, after the model has been fitted. The computation in brms uses Pareto-smoothed importance sampling (PSIS) to approximate the leave-one-out cross-validation efficiently, without needing to refit the model repeatedly. This is done using the pointwise log-likelihood values extracted from the fitted model object and passed to functions in the loo package. The process also includes diagnostics (Pareto k estimates) to check the reliability of the approximation. K-fold: brms provides a dedicated kfold() function, which performs exact K-fold cross-validation by refitting the model multiple times (K times). Users can specify the number of folds, grouping variables, or stratified sampling.

**R2MLwiN**

The R2MLwiN package provides an interface for running the standalone MLwiN software from within R, using either IGLS (maximum likelihood) or MCMC estimation.  WAIC and LOO: These information criteria are not directly or easily computed within the standard R2MLwiN workflow. The package primarily focuses on model fitting and standard model comparisons using likelihood ratio tests or DIC, which is calculated based on the deviance statistic. K-fold: Similar to WAIC/LOO, K-fold cross-validation is not a standard feature of the R2MLwiN package and would require significant manual effort to implement, involving running multiple MLwiN sessions for each fold. The computation of WAIC, LOO, and K-fold cross-validation in R2MLwiN and MCMCglmm varies significantly due to their different estimation approaches and integration with other R packages.  How to compute (external approach): To obtain these measures, users would have to manually extract the log-likelihood values (or predictions) for each observation from the fitted R2MLwiN model (potentially using custom functions) and then use external R packages like the loo package to compute WAIC and LOO. K-fold CV would require manually implementing the data-splitting and model-refitting loop in R, calling R2MLwiN for each fold.

**MCMCglmm**

Estimation Method: MCMCglmm fits generalized linear mixed-effect models using MCMC methods. It provides a full set of posterior samples, which is necessary for the stable computation of WAIC and LOO. WAIC / LOO: MCMCglmm has built-in, though potentially basic, functions or relies on log-likelihood extraction, which can be passed to the highly recommended loo package. The loo package uses Pareto Smoothed Importance Sampling (PSIS) for fast and stable approximation of Leave-One-Out Cross-Validation (PSIS-LOO), which is generally preferred over WAIC. The MCMCglmm package does not have built-in, direct functions for WAIC or LOO, as brms does. WAIC and LOO: Users typically need to manually extract the pointwise log-likelihood from the MCMC samples and then use the loo package functions to compute WAIC or LOO. The package provides deviance values for each iteration, which can be converted to log-likelihood values. The user must write code to create the required S (samples) by N (data points) log-likelihood matrix and pass it to loo::waic() or loo::loo(). K-fold: The package does not have a dedicated kfold() function. Implementing K-fold cross-validation in R requires manual data splitting and model refitting within a user-defined loop.

Computation: The process involves providing the pointwise log-likelihood matrix of the observed data, given the posterior samples, to the loo function. The loo package then computes the expected log predictive density (ELPD), effective number of parameters, and the LOO Information Criterion (LOOIC), along with useful diagnostics (e.g., Pareto k values). If the diagnostics indicate issues, the package may recommend exact LOO-CV for problematic observations. K-fold: Like R2MLwiN, MCMCglmm does not have a single built-in function for automated K-fold CV. How to compute (external approach): K-fold CV must be implemented manually by the user, involving splitting the data into K folds and fitting the MCMCglmm model K times, each time using K-1 folds for training and one fold for validation. Practical application: Despite its limitations, DIC remains a useful tool for practical model comparison in many hierarchical modeling scenarios, especially for ranking models against each other rather than finding a single "correct" model.

A report on PSIS k-diagnostics is included in the updated manuscript as a method for improving the stability of importance sampling estimates by smoothing the importance weights. The stability and reliability of importance sampling estimates, as examined by the Pareto shape parameter, are assessed using PSIS k-diagnostics for LOO within an acceptable range for reliable estimates. A threshold is 0.7.

7. **Numerical issues/table inconsistencies and impossible values.**

Several reported numbers and inconsistencies are concerning: Table 3.1 shows large positive Ultrasound coefficients (4.89) with CI 4.51–9.254 — but Table 3.3 HMC reports Ultrasound Y1 ≈ 0.28 (CI 0.08–0.48). The signs and magnitudes vary across methods far beyond credible intervals. That suggests differences in parameterization, scaling of covariates, or errors in table labeling. Also negative σ_ultrasound of −5.425 (Table 3.1) is impossible for a standard deviation, likely a log-scale parameter or formatting error. Check and confirm.

Number tables according to the PLOS ONE guideline, not as a book chapter or report.

Re-check all tables, standardize variable scaling, explicitly report whether predictors were standardized. Remove or explain impossible negative SDs. Provide posterior density plots for key parameters across methods to show consistency/inconsistency.

**Response:**

R2MLwiN, MCMCglmm, and HMC (Stan) posterior estimates may differ due to the algorithms they use, the different parameterizations defined internally in each R package/algorithm, which have different strengths and weaknesses, as well as different default settings or user-defined choices for things like priors or the data size used. MCMCglmm uses MCMC methods, which can struggle with complex models or correlated posteriors. In contrast, BRMS-Stan uses Hamiltonian Monte Carlo (HMC), which is often more efficient for complex models and can navigate complex posterior landscapes better than Gibbs sampling.  While R2MLwiN relies on specific algorithms tuned for multilevel models that R links to MLwiN, MCMCglmm uses a Gibbs sampler, and BRMS uses a Hamiltonian Monte Carlo (HMC) sampler via Stan, each with distinct convergence properties and performance. Some editorial typos and table labeling errors have been updated.

8. **Diagnostics & posterior predictive checks (PPC) are superficial.**

The paper refers to Figures 3.3–3.7 but there is no informative description of posterior predictive checks, calibration, or residual patterns. Bayesian model assessment must include PPCs tailored to each outcome type (binary outcome calibration plots, ROC/AUC where relevant, continuous residuals). Provide prior/posterior checks, PPC plots and discussion of misfit if any.

Consider including PPC plots (observed vs replicated summary statistics), calibration curves for binary outcome, and a short paragraph interpreting them.

**Response:**

Thank you, dear Reviewer 1. The model diagnostics seem shallow; deeper descriptors are included and updated, including prior predictive checks with default and weakly informative priors. To evaluate a predictive model, PPC plots comparing observed vs. replicated data have been used. A description of posterior predictive checks (PPC) and other trace plots was included in the revised manuscript. The posterior predictive checks show that the model generates replicated data that is reasonably similar to the observed data, as indicated by the overlaid densities (or histograms) of the observed and replicated data.

9. **Use of outdated dataset & clinical context.**

Data were collected in 1988/89: clinical practice and measurement protocols have changed. Discuss this limitation explicitly and do not overclaim clinical implications. Add modern references about ultrasound and cuff pressure diagnostic value for PAD. See <https://doi.org/10.7863/jum.1992.11.3.95> ; and <https://www.ncbi.nlm.nih.gov/books/NBK576430/>

**Response:**

Dear Reviewer 1. We understood your feelings. This small dataset might not be a representative sample for clinical practice and inferences. However, we used it because the study emphasized methodological application across distinct MCMC approaches with no identical priors or likelihood setups. We minimized overclaim clinical implications and inferences in the updated version of the manuscript, based on clinical practice results.

10. **Reproducibility: code and data availability.**

If possible, the manuscript must provide code (Stan/brms/MCMCglmm scripts) and, if patient privacy allows, the data or a simulated dataset reproducing modelling steps. Without code, reviewers cannot verify HW results. Consider adding a GitHub/Zenodo link to code and (if possible) data; if data cannot be shared, provide a simulated example with identical dimensions and signal/noise to reproduce main findings.

**Response:**

The data used and analyzed during the current study are publicly available (published) on Zenodo at the following DOI: <https://doi.org/10.5281/zenodo.17621089>. *To facilitate reproducibility and reuse, all author-generated code used in the manuscript will be made available* ***without restriction*** ***upon acceptance/ publication****. The code will be sent as supplementary material in the final version of the manuscript and will be linked (published) at* [**https://doi.org/10.5281/zenodo.17621419**](https://doi.org/10.5281/zenodo.17621419)**.** *In the meantime, the code is available upon request from the corresponding author via email at* [*end384@gmail.com*](mailto:end384@gmail.com)*. This is due to the project being continuous with the subsequent two phases, which involve related code or data usage.*

11. Ethical Statements: Please, reconsider this statement. Even if your study uses secondary, anonymized, or historical data, most journals require an explicit ethics statement clarifying the ethical status of the data. This ensures transparency and compliance with institutional and journal policies.

**Response:**

We sincerely thank the reviewer for raising this important point of the Ethical statement. However, we thought this study is a modeling application using secondary data from a peer-reviewed, open-access paper. We understand the reviewers' suggestion and have updated it in the revised manuscript, part 2.4

**Additional comments**

(i) Terminology & typos: "Metropolis-Hastening" (multiple occurrences) or "Metropolis–Hastings"? "random gradient" vs "random slope", be consistent. "null (empty) model" is okay but use consistent term (intercept-only). Many grammatical errors and awkward sentences throughout: a thorough language edit is needed.
(ii) Figures & Table formatting: Figures and Tables should be referenced following the journal guidelines. Ensure high-quality figures with captions that describe data plotted and the sample size; tables should include exact definitions of columns (what is ESS, Bulk/Tail ESS, R-hat). Use consistent number of digits.
(iii) If possible, use posterior probabilities or Bayes factors if you want to indicate strength of evidence instead of asterisks for Bayesian credible intervals.
(iv) Matrix notation in Methods mixes many symbols and is hard to read. Provide a short notation table (variables, levels).
(v) Abstract: mention the software used explicitly.

What do you mean by null random intercepts? "models without random effects" or "intercept-only random effects"?

Avoid absolute claims like "yields the most reliable inference" — instead say "provided better performance on the considered information criteria for this dataset" and mention the dataset is small and historical.

Add brief numbers: sample size (N patients, observations).

Remove p-value-style claims in abstract.

(vi) Introduction is well written. But consider structuring it following: (a) joint modeling literature and why joint models help, (b) computational challenges and why algorithm comparison matters, (c) data context and gap the paper fills.

Remove repeated or redundant paragraphs (many paragraphs restate same idea).

Cite and discuss more recent multivariate Bayesian joint-model literature to show novelty <https://doi.org/10.1146/annurev-statistics-112723-034334> <https://doi.org/10.1186/s12874-024-02333-z>

(vii) Methods could be restructured considering (1) the Model specification (explicit mathematical formulation), (2) Priors (Add priors for β (Normal(0, 5)?), for σ (half-Student-t or half-Cauchy?), for correlation matrices (LKJ prior with parameter), and justify choices.)), (3) MCMC & software (software, package versions, and exact settings. For HMC, specify whether models were parameterized centered or non-centered), (4) Model comparison (Explain how WAIC, LOO, K-fold CV, DIC, and ICOMP were computed and which packages computed each. For LOO, report PSIS k estimates and how problematic observations were handled), and (5) Data.
(viii) Results could be organized around Descriptives, Fitted models, Model comparison, and Diagnostic and PPC.
(ix) Discussion and conclusions can be reworked to avoid overstatement. The dataset is small/old, conclusions about clinical practice should be tentative. Emphasize methodological lessons (how HMC handled hierarchical correlation structure when properly parameterized) but include caveats about priors and sample size. Suggest future directions.
(x) For references, add canonical methodological references:

<https://doi.org/10.1186/s12874-024-02333-z> <https://doi.org/10.1146/annurev-statistics-112723-034334> <https://doi.org/10.48550/arXiv.1206.1901> <https://doi.org/10.48550/arXiv.1312.0906> <https://doi.org/10.1007/s11222-016-9696-4>

At present the manuscript cannot be recommended for publication due to the reasons mentioned above. If the authors address the major issues above (particularly full model specification, priors, reproducible code, and consistent results or simulations that explain method differences) and tone down any overstatements about clinical implications, the manuscript could make a useful applied-methods contribution illustrating algorithmic comparisons for joint mixed outcomes. I look forward to see the revised version of this paper.

**Responses to additional comments; reviewer #2**

Thank you for your critical comments. We tried to update the required suggestions and clarifications. Checking whether a Bayesian posterior estimate is significant is based on whether the 95% credible interval includes zero; this is a way to assess its statistical significance. If the interval contains zero, the result is not statistically significant, suggesting there is no evidence of an effect. If the interval does not contain zero, the result is statistically significant.
