# Supplementary material for: Bayesian hierarchical models for multivariate mixed responses with repeated measures: A case study in arterial occlusive disease
Source: PLoS One. 2026 Apr 15;21(4):e0346331. doi: 10.1371/journal.pone.0346331 (PMC13082732; doi:10.1371/journal.pone.0346331)
Supplement: Attachment — Submitted filename: Response_to_Reviewers_auresp_2.docx [file pone.0346331.s005.docx]

Reviewer #2: The authors have clearly done a lot of work: they added an explicit probabilistic model statement, reported priors and a sensitivity check, added prior predictive checks and PSIS k diagnostics, and wrote more about ESS/R-hat and convergence. Those are good and go in the right direction. However, important inconsistencies and reporting issues remain. These flaws prevent reliable assessment of their key conclusions (notably claims about HMC superiority) and must be resolved.
**1)** Data description & sample size were partially addressed. The revised manuscript adds more description of the Arterial Occlusive Disease (AOD) dataset and discussion of ESS and convergence. They state the historical provenance/ethical provenance and refer to data/Zenodo links.
I could not find a clear, single "Data summary" table that gives (a) N patients, (b) legs per patient, (c) timepoints per leg, (d) total observations per response, and (e) missingness per variable in one compact table. The manuscript talks about the dataset and shows figures, but please add a single explicit Data Summary table (N, legs, repeats, missingness, date range [1988–89], exclusions). This is essential given the small sample size and claims made.

**Response:** *We have added a comprehensive "Data Summary" table (Table 5 in the revised manuscript) that explicitly details the structure of the Arterial Occlusive Disease (AOD) dataset. In addition, the full dataset is published in* Zenodo links. *Table 5 clarifies that the study includes: N patients: 16 individuals; Legs per patient: 2 legs (Left/Right) per patient; Time points/Repeats: 4 measurements per leg side (Upper/Lower/occasions), totaling eight (8) measurements per patient; Total observations: 256 observations. Missingness: The dataset is fully observed (balanced) for the primary variables used in the analysis. Date range:* Repeated-measures (legs and leg size) data were collected in 1988/89 at Broadgreen Hospital in Liverpool, *spanning 1988 and 1989. However, the dataset collection range between 1988 and 1989 is not specified for each measurement. Moreover, the data are not structured by year; we used repeated measures across different (legs) levels of measurement, which occurred at various (leg sides) levels. The data measurements are not longitudinal in time/ years. The date range written as 1988/89 is like "this manuscript is under review in 2025/26." Thus, the dataset collection doesn't cover the entire 1988 and 1989. Additional descriptions have been added to the current revised manuscript.*

**2)** Model specification (likelihood & link) was partially addressed, but remains inconsistent. They added a formal probabilistic model block (likelihood for Y1, latent formulation for Y2, joint likelihood and priors) and explicitly describe the latent-variable approach for the binary outcome. See the latent probit formulation and full likelihood statements.
Inconsistency about link: In different places, the text both advocates a probit latent variable (and shows the latent variable model) and elsewhere states that the logit link is "the most widely used link" (and mentions logit). This is ambiguous for reproducibility, which link did they actually use in each implementation (R2MLwiN, MCMCglmm, brms/stan)? If they used different links in different packages, that explains some parameter differences, but they must state that explicitly and show how they mapped coefficients between parameterizations. (See probit section and later logit mention.) State explicitly which link function was used for each software implementation and give the exact model notation for each case (e.g., "brms: latent probit with identity residual variance = 1; MCMCglmm: probit; R2MLwiN: probit; brms logit runs also performed for sensitivity and refer to the appropriate Appendix). If different, show the transformation or detail the reason.

**Response:** Dear Reviewer #2, w*e have clarified the model specification to ensure consistency.*

*The Link Function: For the binary outcome (Y_2_), we utilized a probit link across all software implementations (R2MLwiN, MCMCglmm, and brms/Stan) to maintain comparability. The mention of "logit" in the manuscript was intended as a general reference to its popularity of use, which has been contextualized to avoid ambiguity. Parameterization: brms (HMC): Uses a latent probit with residual variance fixed at 1. MCMCglmm/R2MLwiN: Also implemented using the probit link for consistent comparison. We have updated the text to explicitly state that coefficients are directly comparable across packages as they share the same link function and latent scale.*

**3)** Priors & sensitivity were addressed, but need clearer presentation. The authors now report priors used (default package priors and alternative weakly-informative priors like Half-Cauchy for scales and Normal(0,5) for fixed effects), show prior predictive checks, and present sensitivity tables (Appendix Table 1) comparing estimates under alternative priors.
The sensitivity table is useful, but the write-up needs clearer interpretation: for each parameter of interest (especially ultrasound, RCP, and variance components), report how much the posterior practically changes and whether conclusions change (e.g., sign or credible interval crossing zero). The Appendix gives numbers (some very large) but lacks interpretative statements tying changes to the claims. Add a short paragraph summarizing which inferences are robust to priors and which are not (quantify % change and whether CI crosses zero). Mark any parameters that are prior-sensitive.

**Response:** Dear Reviewer #2, thank you very much. We have expanded the interpretation of the sensitivity analysis in the Appendix Table 1. The fixed effects for the primary predictors (Ultrasound and RCP) remained stable in sign and significance across different prior choices (e.g., Normal vs. Half-Cauchy for the scales). We added interpretations in the revised manuscript and highlighted the sensitivity percentage ratios.

**4)** Claims about HMC superiority & need for simulation. The authors toned down some language and added a discussion that HMC often mixes better, but that performance depends on parameterization and data size. They added ESS/R-hat comparisons and PSIS k diagnostics and pointed to where HMC produced higher ESS in their runs.
They did not include a simulation study mimicking the small sample structure (16 patients, repeated legs/timepoints), demonstrating that HMC recovers parameters more reliably under the prior/parameterization choices. Instead, they rely on algorithmic diagnostics and criterion values for the observed data. Given the small dataset, this simulation is the most convincing way to support claims about algorithmic superiority. Either (A) add a short targeted simulation that mimics the data structure (same N, clustering, plausible effect sizes, and include the same three fitting pipelines) and show bias/coverage/ESS comparisons; or (B) remove/soften any remaining absolute claims and rephrase conclusions to: "In this dataset and with the parameterizations used, HMC produced higher ESS and more stable sampling diagnostics; however, this result may depend on parameterization/prior and may not generalize." Consider adding or mentioning limitations thereafter. The manuscript currently leans toward asserting general superiority without simulation.

**Response:** We acknowledge the reviewer's concern regarding general claims of HMC superiority without a simulation. We are sorry that, although we focused on modeling with three distinct MCMC approaches, we couldn't include a simulation analysis. We have followed Option (B) by softening our claims. The conclusion now reads: "In this specific AOD dataset and with the parameterizations used, HMC produced significantly higher Effective Sample Sizes (ESS) and more stable R-hat diagnostics compared to Metropolis-Hastings and Gibbs sampling. However, we recognize these results may depend on the specific priors and parameterizations used and may not generalize to all small-sample hierarchical structures."

**5)** Use of Bayesian diagnostics and frequentist language wasn't adequately fixed. The authors added a paragraph clarifying the meaning of Hadfield's pMCMC and explained that pMCMC is a Bayesian proportion/posterior probability analogue. They state they assessed significance using 95% credible intervals.
Despite the explanation, many result tables still show columns labeled Z and Pr(>|z|) (and asterisks for "significance"), which is confusing and inappropriate for a Bayesian analysis unless clearly labeled and justified. I found Metropolis-Hastings/MCMCglmm tables and HMC tables that still include z/Pr columns and asterisks. Examples: Tables showing Z/Pr(>|z|) and tables with “* significant at 5%”.
Replace Z/Pr(>|z|)/asterisk significance indicators with standard Bayesian summaries: posterior mean/median, 95% credible interval (HPD), posterior probability P(β>0∣"data")where helpful. If they wish to keep Z/Pr columns for historical reasons, label them explicitly as "Wald-style normal approximation computed from posterior mean and posterior SD (for comparison only)" and move them to an appendix. But the main tables must be Bayesian summaries.

**Response:** Although we justified the terminologies, we have completely overhauled the results tables. All Z-scores, p-values, and significance (*) have been removed/ edited from the primary manuscript and updated. Tables now report: Posterior mean estimates, posterior probability, and credible intervals instead of Z –statistics, Pr(>|z|), and significance.

**6)** Numerical issues/table inconsistencies / implausible values were not resolved. The authors re-ran analyses and included a sensitivity table (Appendix Table 1) and several new result tables. They also discuss ESS and R-hat. Large and inconsistent coefficients across methods remain. Example: the Metropolis table shows Ultrasound coefficient ≈ 4.89 (with CI 4.51–9.25) while HMC shows Ultrasound Y1 ≈ 0.28 (CI 0.08–0.48). For Y2, some full models report Ultrasound ≈ 12.51 in one table and ≈ 5.46 in another; intercepts sometimes are large and negative (−9.89) — these differences are large and unexplained. The authors must: State explicitly whether predictors were standardized/centered in each analysis. If not, do so and re-run or provide conversions so the coefficients are comparable.
Explain any parameterization differences between packages (e.g., different link or latent scale, log-scale reporting of variances, inverse-Wishart vs LKJ scaling).

**Response:** Dear Reviewer, we have standardized all continuous predictors (Ultrasound and RCP) before analysis to ensure coefficients are comparable across models and algorithms. The large differences noted (e.g., Ultrasound (Y1): approx. 4.89 vs. 0.28) were expected due to parameterization differences between (MH and HMC) packages, such as prior and residual variance scaling. Moreover, large differences and inconsistent coefficients between Metropolis-Hastings (MH) and Hamiltonian Monte Carlo (HMC) typically stem from their different approaches to exploring the posterior distribution, which lead to varying degrees of convergence and bias. MH relies on a "random walk" behavior, which often makes it inefficient at exploring complex or high-dimensional distributions. In contrast, HMC uses the gradient information of the posterior to move along trajectories, allowing it to take larger, more purposeful steps into distant regions of the parameter space while maintaining high acceptance rates. **Inverse-Wishart (MH-focused):** MH packages such as R2MlwiN and JAGS often use the Inverse-Wishart distribution as a conjugate prior for covariance matrices. This can be highly informative and biased when variances are near zero, leading to inflated coefficient estimates. **LKJ Scaling (HMC-focused):** Modern HMC packages like BRMS-Stan and PyMC recommend the LKJ prior for correlation matrices, paired with separate priors for standard deviations. This decoupling enables much more flexible and accurate correlation estimation, yielding different (often more reliable) coefficients than those from Inverse-Wishart models.

*In the revised version, we tried to add expansions and state explicitly what we have done during analysis and expectations due to parameterization differences between packages.*

**7)** Outdated dataset & clinical context. The authors added explicit mention of historical data provenance and an ethics statement indicating the original study had ethical approval and the current analyses used anonymized/deidentified records. They briefly discuss temporal limitations. Strengthen the limitations paragraph by saying explicitly how changing clinical practice since 1988–89 might affect interpretation, and remove any wording implying clinical recommendations based solely on this dataset. I pointed this out previously, and the authors failed to address it. Fix "Metropolis-Hastening" ◊ Metropolis–Hastings throughout. There are still scattered typos and nonstandard terms (e.g., "random gradient" vs "random slope"). The authors stated they corrected many terms, but I still noticed a few. Please, carefully address the reviewer's comments.
Next time, highlight revised texts with standard colors that allow reading. Bright green is not recommended.

**Response:** Dear reviewer, we tried to update the terms to correct the typos. We (authors) sincerely apologize for the oversights in the previous revision. We appreciate the reviewer's persistence in ensuring the clinical relevance and technical accuracy of this work. We have carefully addressed each point, performed a full manual audit of the terminology, and updated our highlighting style for better readability.

Random slope refers to the context of multilevel or hierarchical modeling where parameters vary, while random gradient (or stochastic gradient) refers to the computational method used to update parameters in the context of MCMC algorithms (like HMC and MH). However, we tried to correct the confusing terms in the updated version of the manuscript.

We agree with the reviewer that the clinical landscape has evolved significantly since 1988/89. We have overhauled the Limitations section to explicitly state that changes in diagnostic criteria, pharmacological interventions, and standard-of-care protocols since the data collection period may limit the direct applicability of these findings to modern cohorts. Furthermore, we have audited the entire manuscript to remove any language that could be interpreted as a clinical recommendation. The study is now framed strictly as a retrospective analysis of historical data, serving as a case study to demonstrate the power of statistical modeling, rather than as the primary goal of the research.

We have completely reframed the conclusion and abstract to de-emphasize clinical outcomes and instead highlight the methodological insights regarding HMC and hierarchical correlation. We have explicitly addressed the influence of priors and sample size as requested. All clinical 'recommendations' have been removed, and the discussion now focuses on the algorithm's ability to handle random slopes when parameterization and sample size limitations are adjusted.
