# Supplementary material for: Bayesian hierarchical models for multivariate mixed responses with repeated measures: A case study in arterial occlusive disease
Source: PLoS One. 2026 Apr 15;21(4):e0346331. doi: 10.1371/journal.pone.0346331 (PMC13082732; doi:10.1371/journal.pone.0346331)
Supplement: Attachment — Submitted filename: Response_to_Reviewers_auresp_3.docx [file pone.0346331.s006.docx]

**Journal Requirements:**

1. If the reviewer comments include a recommendation to cite specific previously published works, please review and evaluate these publications to determine whether they are relevant and should be cited. There is no requirement to cite these works unless the editor has indicated otherwise.

**Response:** It is okay. We have no reviewers’ comments to include additional references.

2. Please review your reference list to ensure that it is complete and correct. If you have cited papers that have been retracted, please include the rationale for doing so in the manuscript text, or remove these references and replace them with relevant current references. Any changes to the reference list should be mentioned in the rebuttal letter that accompanies your revised manuscript. If you need to cite a retracted article, indicate the article’s retracted status in the References list and also include a citation and full reference for the retraction notice.

**Responses:** We have thoroughly audited our reference list to ensure all citations are complete, accurate, and current. We have specifically verified the status of all cited works against retraction databases and confirm that no retracted articles are included in this revision. Furthermore, we have cleaned all DOI links, removing extraneous metadata tags (e.g., /BIBTEX, /S1) to ensure direct and uninterrupted access for readers, as commented by Reviewer #2.”

**Reviewer #2: PONE-D-25-42936R2 Comment**

The authors have made substantial and constructive revisions that have significantly improved the overall quality, clarity, and technical rigor of the manuscript. The responses to prior comments are generally appropriate and demonstrate careful consideration of the review process. Nevertheless, a small number of minor issues remain that should be addressed to ensure consistency, precision, and completeness before a final decision can be reached. Specific recommendations for these final minor adjustments are provided below.
1. Terminology and abbreviations must be introduced once at first occurrence and then used consistently without redefinition throughout the manuscript. This is critical in the manuscript, where repeated expansion of the same term disrupts readability and suggests insufficient editorial control. One example is the expression “Hamiltonian Monte Carlo (HMC)” that is constantly defined throughout the manuscript. For example, the paragraph after Table 3 starts and ends with “Hamiltonian Monte Carlo (HMC)”, followed by Table 4, which again reintroduces “Hamiltonian Monte Carlo (HMC)” as though it had not been defined earlier.
Consistent abbreviation usage will improve flow, reduce redundancy, and enhance the professional presentation of the manuscript. This should be revised throughout the manuscript for all other terms as well.

**Response:** We thank the reviewer for this observation. We have conducted a thorough review of the manuscript to ensure that all abbreviations, including Hamiltonian Monte Carlo (HMC), Metropolis-Hastings (MH), Arterial Occlusive Disease (AOD), Effective Sample Size (ESS), and Markov Chain Monte Carlo (MCMC), are defined only at their first occurrence in the Abstract and the first mention in the Introduction. Subsequent mentions have been replaced with the abbreviated forms to improve readability and eliminate redundancy as requested.

2. Captions could be improved. For example, the caption of Table 9 is not self-contained.
**Response:** We agree that table captions should be self-explanatory. We have revised the caption for Table 9 to include a comprehensive description of the metrics (DIC, ICOMP, WAIC, LOO-IC, and K-fold) and the specific models being compared (null, random-intercept, and full random-slope). This ensures the table is fully interpretable without immediate reference to the main text.”

3. Please standardize the notation used for figure references throughout the manuscript. Both “Fig.” and “Fig” are currently used; only one format should be adopted consistently in accordance with the journal style guide (typically “Fig.” with a period). Consistent formatting contributes to a more professional and polished presentation.
Response: We apologize for the inconsistency in figure citations. In accordance with the journal’s style guide, we have standardized all figure references to the correct format for figure labels, which is “Fig” followed by an Arabic numeral without a period (e.g., Fig 1, Fig 2, Fig 3) throughout the entire manuscript. We also reformatted the in-text citations to use the format “Fig 1”, “Fig 2”, or “Figs 1 and 2”.

4. Figure citation order in the main text must follow a strictly sequential progression. At present, the narrative flow is disrupted because the discussion moves from Fig. 4 directly to Fig. 9, while Figs. 5–8 are introduced later or not at all. Specifically, Fig. 9 appears in the second paragraph of Section 3.4 (“Evaluation of MCMC convergence diagnostics and conditional/marginal effects for the best-fitted model”), whereas Figs. 5–7 are first cited approximately four pages later (paragraph 3), and Fig. 8 does not appear to be referenced in the text. Figures should be introduced in numerical order (4 > 5 > 6 > 7 > 8 > 9) at the point where their content is first discussed. This reordering is essential to maintain logical continuity and to help readers follow the progression of results without confusion.

**Response:** We have restructured the manuscript to ensure that all figures are introduced in a strictly sequential numerical order. Figures 5 through 8 have been repositioned or re-cited in the narrative to precede the discussion of Fig. 9 in Section 3.4, ensuring a logical flow of results for the reader.

5. Revise the DOI to references and make sure they are all accessible to the readers of the paper. Some are below (I limit the list below to letters A-D only), just to show how critical this issue is.
i) Kallioinen, N., Paananen, T., Bürkner, P. C., & Vehtari, A. (2024). Detecting and diagnosing prior and likelihood sensitivity with power-scaling. Statistics and Computing, 34(1), 1– 27. <https://doi.org/10.1007/S11222-023-10366-5/FIGURES/1>
ii) Brooks, S., Gelman, A., Jones, G. L., & Meng, X. L. (2011). Handbook of Markov Chain Monte Carlo. Handbook of Markov Chain Monte Carlo, 1–592. <https://doi.org/10.1201/B10905/HANDBOOK-MARKOV-CHAIN-MONTE-CARLO> GALIN-JONES-XIAO-LI-MENG-ANDREW-GELMAN-STEVE-BROOKS/RIGHTS AND-PERMISSIONS
iii) Chien, Y. F., Zhou, H., Hanson, T., & Lystig, T. (2023). Informative g-Priors for Mixed Models. Stats, 6(1), 169–191. <https://doi.org/10.3390/STATS6010011/S1> (remove “/S1”)
iv) Cook, S. R., Gelman, A., & Rubin, D. B. (2006). Validation of software for Bayesian models using posterior quantiles. Journal of Computational and Graphical Statistics, 15(3), 675–692. <https://doi.org/10.1198/106186006X136976;REQUESTEDJOURNAL:JOURNAL:UCG> S20;WGROUP:STRING:PUBLICATION (correct = <https://doi.org/10.1198/106186006X136976> )
v) Depaoli, S., Winter, S. D., & Visser, M. (2020). The Importance of Prior Sensitivity Analysis in Bayesian Statistics: Demonstrations Using an Interactive Shiny App. Frontiers in Psychology, 11, 608045. <https://doi.org/10.3389/FPSYG.2020.608045/BIBTEX> (correct is <https://doi.org/10.3389/fpsyg.2020.608045> )

**Response:** We appreciate the reviewer’s meticulous check of our references. We have corrected all DOIs in the reference list, removing unnecessary suffixes and ensuring they point directly to the source articles/ books. We have verified each link for accessibility. We also formatted using the PLOS One reference style.
