# Supplementary material for: Bayesian hierarchical models for multivariate mixed responses with repeated measures: A case study in arterial occlusive disease
Source: PLoS One. 2026 Apr 15;21(4):e0346331. doi: 10.1371/journal.pone.0346331 (PMC13082732; doi:10.1371/journal.pone.0346331)
Supplement: Attachment — Submitted filename: Response_to_Reviewers_auresp_4.docx [file pone.0346331.s007.docx]

**Dear Reviewer #2 and the Editor,**

Thank you for your constructive and thorough review of our manuscript. We have carefully addressed each of your [editor] comments to improve the clarity, professionalism, and accuracy of our work. Please find our point-by-point responses below.

**Journal Requirements:**

1. If the reviewer comments include a recommendation to cite specific previously published works, please review and evaluate these publications to determine whether they are relevant and should be cited. There is no requirement to cite these works unless the editor has indicated otherwise. 
Response: There is no reviewer comment to include a specific published work.

2. Please review your reference list to ensure that it is complete and correct. If you have cited papers that have been retracted, please include the rationale for doing so in the manuscript text, or remove these references and replace them with relevant current references. Any changes to the reference list should be mentioned in the rebuttal letter that accompanies your revised manuscript. If you need to cite a retracted article, indicate the article’s retracted status in the References list and also include a citation and full reference for the retraction notice.

Response: We have checked all the references, and all are correct

**Reviewer #2:** I appreciate the authors’ effort in revising the manuscript and their willingness to address the previous comments. The work has improved, and I would like to see it progress further toward acceptance. However, several minor issues remain and should be carefully addressed to ensure the manuscript meets the expected standards of scientific presentation and editorial quality.
First, the citation format within the text requires systematic correction. There should be a space between the preceding word and the citation number. For example, “structures[7]” should be revised to “structures [7].” This formatting issue appears throughout the manuscript and should be corrected consistently.

**Response:** We apologize for this oversight, which occurred due to the automatic Mendaly update citation. We have conducted a systematic review of the entire manuscript and corrected the formatting to ensure a space consistently precedes all citation brackets.

Second, the narrative integration of references is often inappropriate. Expressions such as “[11] used R” or “. [19] demonstrated the usefulness” are not acceptable in standard scientific writing. Instead, references should be incorporated into the sentence in a grammatically correct manner by naming the authors, followed by the citation number. For example, this should be written as “Lemoine (2019) used R … [11].” or “Tate and Pituch (2007) demonstrated the usefulness … [19].” This improves readability, attribution clarity, and professionalism. The authors should revise the manuscript throughout to ensure all references are introduced properly.

**Response:** We have revised the narrative throughout the manuscript to properly introduce references using author names followed by the citation number, improving the flow and professional tone of the writing.

Third, and most concerning, the DOI and reference corrections requested previously have not been adequately implemented. Although the authors indicated that these had been revised, numerous references in the revised manuscript still contain incorrect, duplicated, or non-functional DOI links. This suggests insufficient attention during revision. Correct and accessible DOIs are essential to allow readers to verify and consult the cited literature. The following critical issues must be corrected:
• Reference [12]: Incorrect DOI. The correct DOI is: <https://doi.org/10.1515/jqas-2018-0106>
• Reference [13]: Incorrect DOI. The correct DOI is: <https://doi.org/10.1186/s12874-024-02333-z>
• References [30] and [31]: Incorrect DOI, same issue as [12]
• References [40] and [55]: Incorrect DOI, same issue as [12]
• References [65], [66], and [78]: Incorrect DOI, same issue as [12]
• Reference [41]: Duplicate DOI
• Reference [53]: Same duplicated DOI as [41]
• Reference [60]: DOI and reference details require verification and correction
The authors should carefully review the entire reference list, verify each DOI individually, ensure that each DOI points to the correct article, remove duplicate entries, and confirm that all references are accurate and accessible.

**Response:**

12. <https://doi.org/10.1515/jqas-2018-0106>

13. <https://doi.org/10.1186/s12874-024-02333-z>

30. <https://doi.org/10.1201/b10905>

31. <https://doi.org/10.3758/s13423-016-1221-4>

40. <https://doi.org/10.1186/s40623-022-01645-y>

41. <https://doi.org/101214/20-BA1246>

53. <https://doi.org/10.1214/19-STS731>

66. <https://doi.org/10.3389/fpsyg.2020.608045>

71. <https://doi.org/10.3390/stats6010011>

etc…

Previous comment #1: stating "Terminology and abbreviations must be introduced once at first occurrence and then used consistently without redefinition throughout the manuscript. This should be revised throughout the manuscript for all other terms as well."
The manuscript does not follow standard conventions regarding the introduction and consistent use of abbreviations. Terminology and abbreviations should be defined at their first occurrence and used consistently thereafter without redefinition. This issue persists despite my previous request for correction. In particular, the abbreviation MCMC is used in the Abstract without definition, appears again in the first paragraph of the introduction, and is only defined later in the fourth paragraph of that section. This delayed definition disrupts readability and forces the reader to proceed without knowing the meaning of a key methodological term. The current structure is therefore awkward and should be revised so that MCMC is defined at its first occurrence in the Abstract and then used consistently throughout the manuscript. Similar attention should be applied to all other abbreviations to ensure clarity and proper scientific presentation.

**Response:** We thank the Reviewer for pointing out the inconsistency in our use of abbreviations. We were experienced in defining abbreviations only at their first appearance in the main text (usually the introduction), regardless of whether they were defined in the Abstract. We have performed a thorough review of the manuscript to ensure that all technical terms and abbreviations, such as **MCMC**, **HMC**, and **ESS**, are defined at their first occurrence in the text and used consistently thereafter without redefinition. We have also removed redundant definitions in the Materials and Methods and Results sections to adhere to standard scientific writing conventions.
